# Maternal Serum High-Sensitivity C-Reactive Protein (hsCRP) as a Prognostic Marker of Fetomaternal Outcome in Hypertensive Disorders of Pregnancy: A Novel Study

**DOI:** 10.7759/cureus.24327

**Published:** 2022-04-20

**Authors:** Ketav Joshi, Neema Acharya, Sourya Acharya, Samir Joshi

**Affiliations:** 1 Department of Obstetrics and Gynaecology, Jawaharlal Nehru Medical College, Datta Meghe Institute of Medical Sciences, Wardha, IND; 2 Department of Medicine, Jawaharlal Nehru Medical College, Datta Meghe Institute of Medical Sciences, Wardha, IND; 3 Department of Obstetrics and Gynaecology, Dr. Vinod Joshi Maternity Nursing Home, Mumbai, IND

**Keywords:** foetal outcome, maternal outcome, high-risk pregnancy, hypertensive states of pregnancy, hscrp

## Abstract

Introduction: Hypertensive disorders of pregnancy (HDP) are a group of obstetric disorders causing profound fetomaternal compromise, leading to adverse obstetric outcomes. High-sensitivity c-reactive protein (hsCRP), an inflammatory marker of systemic inflammation, is elevated in HDP and correlates with the severity of the disease. However, prediction and prevention of HDP and its associated fetomaternal complications remain elusive to most obstetricians. The present study aimed to evaluate the use of hsCRP as a prognostic marker of adverse fetomaternal outcome in HDP.

Methods: The study included 132 third-trimester pregnancies with HDP who underwent hsCRP quantification at the time of presentation to the out-patient department and followed up till delivery. HsCRP quantification was done using immunoturbidimetry method.

Results: Of the 132 cases studied, 72 had normal hsCRP levels while the remaining 60 had raised hsCRP levels. It was observed that patients with raised hsCRP levels had poorer fetomaternal outcomes at delivery as compared to those with normal hsCRP levels.

Conclusion: The obstetric outcomes of patients with HDP worsened with increasing levels of hsCRP, as shown in our study, when compared to normotensive patients. Thus, hsCRP delivers promising results as a prognostic marker of adverse fetomaternal outcomes in patients of HDP.

## Introduction

Epidemiological studies done on maternal and child health (MCH) document the increase in the incidence of hypertension in pregnancy and account for more than 50,000 deaths worldwide annually [1]. Hypertensive disorders of pregnancy and their associated fetomaternal complications are the leading conditions to cause increased fetomaternal morbidity and mortality [2]. Despite the government’s initiatives and various programs on maternal and child health care, prediction and prevention of hypertensive disorders in pregnancy remains elusive to most obstetricians. Though there have been advances in the prediction and management protocols for hypertension in pregnancy, its complications are unavoidable in most cases. Numerous factors like hypoxia, angiogenic factors, impaired immunity, and inflammatory cascades are implicated in the occurrence of hypertension in pregnancy and its complications [3].

Hypertensive disorders of pregnancy often culminate in adverse obstetric and neonatal outcomes, and thus, there is a need for early detection and effective management. The present study aimed to investigate and discuss the role of hsCRP as a prognostic marker of hypertensive disorders of pregnancy and the associated fetomaternal outcome.

Pathophysiology and classification of hypertensive disorders in pregnancy

According to the American College of Obstetricians and Gynaecologists (ACOG), gestational hypertension and pre-eclampsia have preponderance over other hypertensive disorders of pregnancy [4]. They further deteriorate into eclampsia when a convulsion develops or may manifest as hemolysis, elevated liver enzymes, and low platelet count (HELLP) syndrome. Eclampsia and HELLP syndrome are associated with severe complications such as cerebral hemorrhage, liver hemorrhage, lung edema, and renal insufficiency [5]. Maladaptation of maternal immune responses and defective trophoblast invasion are hypothesized in the etiology of pre-eclampsia. Thus, an excessive maternal inflammatory response ensues against foreign fetal antigens and triggers a chain of events that include the release of pro-inflammatory cytokines in the systemic circulation, defective spiral artery remodeling, abnormal trophoblast invasion, and placental infarcts which lead to poor fetal outcome [6].

## Materials and methods

The present study was conducted in the Department of Obstetrics and Gynaecology at Datta Meghe Institute of Medical Sciences (DMIMS), Wardha, over a span of three years. The Institutional Ethics Committee of DMIMS approved the study protocol and informed consent of all participants was obtained. The study consisted of 132 cases of hypertensive disorders of pregnancy (HDP) at ≥28 weeks of gestation who underwent hsCRP evaluation at the time of diagnosis of HDP and were followed up till delivery. General, systemic and obstetric examination was done at admission and the diagnosis of HDP was made according to the ACOG guidelines, 2020 [7]. A summary of the classification of hypertensive disorders of pregnancy by various academic societies is shown in Table 1 [8]. 

**Table 1:** Classification of hypertensive disorders of pregnancy by various academic societies JSSHP: Japan Society for the Study of Hypertension in Pregnancy; ISSHP: International Society for the Study of Hypertension in Pregnancy; ACOG: American College of Obstetricians and Gynecologists; SOGC: Society of Obstetricians and Gynecologists of Canada; SOMANZ: Society of Obstetric Medicine of Australia and New Zealand; NHBPEP: National High Blood Pressure Education Program

JSSHP (HRP, 2013)	ISSHP (A revised statement from the ISSHP, 2014)	ACOG (Task Force on Hypertension in Pregnancy, 2013)	SOGC (Working Group, 2014)	SOMANZ (Guidline, 2014)	NHBPEP (Working Group on High Blood Pressure in Pregnancy, 2000)
Pregnancy-induced hypertension (PIH)	Hypertensive disorders of pregnancy	Hypertensive disorders of pregnancy	Hypertensive disorders of pregnancy	Hypertensive disorders of pregnancy	Hypertensive disorders of pregnancy
Gestational hypertension (GH)	Gestational hypertension	Gestational hypertension	Gestational hypertension · With comorbid condition(s) · With evidence of preeclampsia	Gestational hypertension	Gestational hypertension
Preeclampsia (PE)	Preeclampsia de novo	Preeclampsia-eclampsia	Preeclampsia	Preeclampsia-eclampsia	Preeclampsia-eclampsia
Eclampsia (E)					
Superimposed preeclampsia (S-PE)	Superimposed preeclampsia on chronic hypertension	Chronic hypertension with superimposed preeclampsia	Chronic hypertension with superimposed preeclampsia	Preeclampsia superimposed on chronic hypertension	Preeclampsia superimposed on chronic hypertension
Chronic hypertension	Chronic hypertension	Chronic hypertension	Chronic hypertension with or without comorbid conditions	Chronic hypertension with its variants such as essential hypertension, secondary hypertension, or white coat hypertension	Chronic hypertension
Appendix	White coat hypertension	-	Other hypertensive effects such as transient hypertensive effect, white coat hypertension, or masked hypertensive effect		-
-	-	Postpartum hypertension	-	-	-

Pregnant women with BMI < 18 kg/m^2^ or BMI > 25 kg/m^2^, history of acute or chronic systemic inflammatory conditions, diabetes mellitus or gestational diabetes, or major fetal anomalies were excluded from the study. A prestructured proforma was used to collect the data. A 10 mL of 12-hour overnight fasting venous blood sample was collected from patients for hsCRP estimation along with haemogram, hepatic and renal function tests, prothrombin time, and international normalized ratio (INR) analysis. Estimation of hsCRP was done by immunoturbidimetry method and the normal range accepted was 0.1-3.0 mg/L [9]. The patients were divided into group A (normal hsCRP levels) and group B (raised hsCRP levels) having 72 and 60 patients, respectively. Subjects were managed as per departmental protocols and regular monitoring was done to ensure maternal and fetal well-being. The patients were followed-up till delivery and numerous maternal and fetal outcomes were observed in both the study groups. Chi-square test and student t-test were used to find the significance of various parameters of the study.

## Results

A total of 132 patients participated in the study. Seventy-two patients had normal hsCRP levels while 60 patients had raised hsCRP. The observations of the study are summarized as follows: the various parameters observed during antenatal fetomaternal monitoring are presented in Table 2. P-value <0.01 was considered statistically significant.

**Table 2:** Summary of observed parameters during antenatal and fetomaternal monitoring between both groups *Statistically significant. AST: aspartate aminotransferase; ALT: alanine aminotransferase; LDH: lactate dehydrogenase; S/D ratio: systolic/diastolic ratio; hsCRP: high-sensitivity c-reactive protein

Study parameters	Normal hsCRP (N=72)	Raised hsCRP (N=60)	p-Value
hsCRP levels (mg/L)	2.31 ± 0.45	5.85 ± 1.82	<0.01*
Age (years)	24.71 ± 3.42	26.35±5.05	0.19
Parity (primigravida)	34 (47.20%)	33 (55.00%)	0.42
Systolic blood pressure (mmHg)	144.03 ± 5.73	151.33 ± 13.59	<0.01*
Diastolic blood pressure (mmHg)	92.78 ± 4.81	97.00 ± 7.66	<0.01*
Haemoglobin (g/dL)	11.50 ± 1.19	10.54 ± 1.71	<0.01*
Platelet count (×10^5^/mL)	2.23 ± 0.65	1.61 ± 0.62	<0.01*
Serum bilirubin (mg/dL)	0.56 ± 0.16	0.74 ± 0.50	0.004*
Serum AST (U/L)	17.32 ± 5.49	23.10 ± 16.89	0.007*
Serum ALT (U/L)	27.42 ± 6.48	41.42 ± 37.79	0.002*
Serum total proteins (g/dL)	7.32 ± 0.66	6.90 ± 0.72	0.03
Serum LDH (U/L)	297.43 ± 57.13	453.62 ± 343.34	<0.01*
Serum creatinine (mg/dL)	0.54 ± 0.32	0.63 ± 0.94	0.039
Serum urea (mg/dL)	7.97 ± 1.96	9.52 ± 2.97	0.027
Prothrombin time (s)	12.36 ± 0.32	12.77 ± 0.94	0.001*
International normalized ratio (INR)	1.02 ± 0.013	1.05 ± 0.069	0.001*
Liquor index on ultrasound	11.18 ± 3.87	7.38 ± 4.15	0.001*
Abnormal Doppler studies (S/D ratio)	2 (2.78%)	17 (28.33%)	<0.01*
Fetal growth restriction	1 (1.39%)	28 (46.67%)	<0.01*

There was a statistically significant difference between hsCRP levels of participants of both groups, as depicted in Table 2. The results state that a positive statistical correlation was observed in systolic and diastolic blood pressures, mean values of serum bilirubin, aspartate aminotransferase (AST), serum alanine aminotransferase (ALT), lactate dehydrogenase (LDH), prothrombin time (PT), international normalized ratio (INR), fetal ultrasound Doppler studies (S/D ratio), fetal growth restriction, distribution of clinical presentation of hypertensive disorders of pregnancy (HDP), and mode of delivery between both the study groups. There was a negative correlation between mean levels of serum hemoglobin, platelet count, and mean liquor index on ultrasonography between participants of both groups. However, no statistical significance was observed in the age, parity, mean serum total proteins, serum creatinine, and serum urea between both the study groups.

Table 3 summarizes the distribution of HDP among patients of both groups. There was a statistically significant correlation (p-value <0.01) between clinical presentations of HDP in both the study groups. Most of the patients with normal hsCRP levels presented with gestational hypertension while a greater fraction of patients with raised hsCRP levels presented with severe preeclampsia. There were no cases of chronic hypertension or superimposed preeclampsia in the study period. 

**Table 3:** Summary of distribution of hypertensive disorders of pregnancy between both groups *Statistically significant. HsCRP: high-sensitivity c-reactive protein

Study parameters	Normal hsCRP (N=72)	Raised hsCRP (N=60)	p-Value
Gestational hypertension	40 (55.56%)	17 (28.33%)	<0.01*
Mild preeclampsia	28 (38.89%)	19 (31.67%)	
Severe preeclampsia	3 (4.17%)	21 (35.00%)	
Eclampsia	1 (1.39%)	3 (5.00%)	

Table 4 states that there was a statistically significant in the mode of delivery between both the groups (p-value <0.01). It was observed that patients with raised hsCRP levels formed a greater fraction of all cesarean deliveries. Cesarean section also proved to be the most frequent mode of termination of pregnancy among patients with raised hsCRP levels. 

**Table 4:** Summary of distribution of mode of delivery between both groups *Statistically significant. HsCRP: high-sensitivity c-reactive protein

Study parameters	Normal hsCRP (N=72)	Raised hsCRP (N=60)	p-Value
Vaginal delivery	64 (88.89%)	10 (16.67%)	<0.01*
Instrumental vaginal delivery	3 (4.17%)	6 (10.00%)	
Cesarean section	5 (6.94%)	44 (73.33%)	

Table 5 compares the incidence of maternal complications between both the study groups. There was a statistically significant difference in the incidence of acute liver injury (ALI) and hemolysis, elevated liver enzymes, and low platelet count (HELLP) syndrome while no statistical difference was observed in the incidence of acute kidney injury (AKI), disseminated intravascular coagulopathy (DIC), and neurological manifestations (such as intracranial hemorrhage or posterior reversible encephalopathy {PRES} syndrome) between participants of both study groups. There was also a difference in the incidence of abruptio placentae between both groups though it did not reach statistical significance. There was no maternal mortality in either group. 

**Table 5:** Summary of maternal complications between both groups *Statistically significant. HELLP: hemolysis, elevated liver enzymes, and low platelet count; hsCRP: high-sensitivity c-reactive protein

Study parameters	Normal hsCRP (N=72)	Raised hsCRP (N=60)	p-Value
Abruptio placentae	0 (0.00%)	5 (8.33%)	0.013
Acute liver injury	0 (0.00%)	18 (30.00%)	<0.01*
Acute kidney injury	1 (1.39%)	3 (5.00%)	0.23
Disseminated intravascular coagulopathy	0 (0.00%)	4 (6.67%)	0.22
HELLP syndrome	1 (1.39%)	12 (20.00%)	<0.01*
Neurological symptoms (including eclampsia)	1 (1.39%)	4 (6.67%)	0.16

Table 6 compares the incidence of fetal and neonatal complications between both the study groups. There was a positive statistical correlation in the incidence of non-stress test (NST) abnormalities, meconium-stained liquor (MSL), low birth weight, preterm delivery, neonatal intensive care unit (NICU) admission rates and intrauterine fetal demise (IUFD), and neonatal mortality in NICU between both the study groups. There were no stillbirths during the study.

**Table 6:** Summary of fetal complications between both groups *Statistically significant. NICU: neonatal intensive care unit; hsCRP: high-sensitivity c-reactive protein

Study parameters	Normal hsCRP (N=72)	Raised hsCRP (N=60)	p-Value
Non-stress test (NST) abnormalities	1 (1.39%)	42 (70.00%)	<0.01*
Meconium-stained liquor	1 (1.39%)	25 (41.67%)	<0.01*
Birth weight <2500g	2 (2.78%)	45 (75.00%)	<0.01*
Gestational age <37 weeks	3 (4.16%)	32 (53.33%)	<0.01*
NICU admission	1 (1.39%)	26 (43.33%)	<0.01*
Intrauterine fetal demise	0 (0.00%)	5 (8.33%)	<0.01*
Neonatal mortality	0 (0.00%)	5 (8.33%)	<0.01*

**Figure 1:**
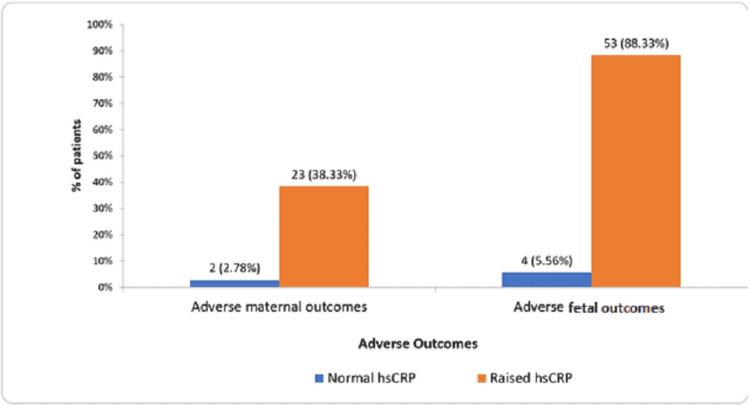
Graph depicting the incidence of maternal and fetal complications between participants of both groups HsCRP: high-sensitivity c-reactive protein

Figure 1 depicts the incidence of adverse maternal and fetal outcomes between participants of both groups in terms of complications during the course of pregnancy. It was observed that while only two (2.78%) patients with normal hsCRP levels suffered from one or more maternal complications, the incidence rose to 23 (38.33%) patients in the study group with raised hsCRP levels. Similarly, the incidence of fetal and neonatal complications in patients with normal and raised hsCRP levels was four (5.56%) and 53 (88.33%), respectively.

## Discussion

Hypertensive disorders of pregnancy (HDP) are a group of syndromes with systemic manifestations and occur globally in 3-5% of pregnant women, clinically presenting after 20 weeks of gestation as new-onset hypertension with or without proteinuria, a major contributor to maternal and neonatal morbidity and mortality [10]. Identifying pregnancies with higher risk of HDP is considered a major challenge in obstetric science. Early detection of the disorder would allow early interventions, so as to improve placental milieu and reduce the occurrence of HDP [11]. HDP are characterized anatomically by inappropriate remodeling of spiral arteries of the placenta, occurring after 20 weeks of gestation with widespread vascular endothelial malfunction and vasospasm [12]. The exact pathophysiology of the development of preeclampsia is not well understood, though it is hypothesized to be primarily by this abnormal vascular response to inappropriate placentation, often detected only after significant pathological changes have set in and this restricts the obstetrician to limited treatment options [13,14]. Therefore, primary screening, early diagnosis, and intensive management of HDP are paramount to improve fetomaternal outcomes. If they can be diagnosed incipiently, high-risk patients can benefit from intensive obstetric care and this could improve maternal and fetal prospects. As hsCRP is known to be a marker of systemic inflammation in pregnancy, it could play a crucial role in the early detection of HDP.

In the present study, the patients were of comparable age and parity. There was no maternal mortality or stillbirths during the study period. Patients with raised hsCRP levels had higher mean systolic and diastolic blood pressures as compared to patients with normal hsCRP levels. The biochemical and radiological parameters also varied between participants of both groups with poorer results in patients having raised hsCRP (Table 2). Patients with raised hsCRP levels showed higher incidence of severe preeclampsia and eclampsia (Table 3). Consequently, they presented with a higher rate of maternal and fetal complications, as depicted in Tables 5, 6 and Figure 1. These patients had a higher rate of cesarean sections due to fetal heart rate abnormalities (NST) (Table 4). They consistently demonstrated a higher incidence of obstetric complications and ultimately poorer fetomaternal outcomes.

Numerous studies have elicited a significant correlation between hsCRP and the development of preeclampsia but present evidence is deficient in demonstrating its potential as a prognostic marker in HDP or comparing different clinical presentations of HDP. The present study is a novel project in this interest as it encompasses both of these deficiencies. Table 7 compares the findings of various studies undertaken to analyze the role of hsCRP in prediction of preeclampsia with the findings of the present study. It was observed that the bulk of the studies found positive correlation between hsCRP levels of their respective study groups.

**Table 7:** Summary of various studies on hsCRP as a predictive marker of preeclampsia and comparison with the present study *Median (interquartile range). AST: aspartate aminotransferase; ALT: alanine aminotransferase; LDH: lactate dehydrogenase; hsCRP: high-sensitivity c-reactive protein; HDP: hypertensive disorders of pregnancy; SBP: systolic blood pressure; DBP: diastolic blood pressure

HsCRP levels in study (Mean±SD)	Normotensive patients	Patients with mild preeclampsia	Patients with severe preeclampsia	Statistical inference
Sayyad and Pratinidhi, 2020 [15]	1.47 ± 0.59	2.89 ± 1.12	4.3 ± 0.58	Correlation between hsCRP, SBP, and DBP.
Mishra et al. 2019 [16]	2.25 (1.1‑7.6)*	5.3 (2.8 12.4)*	6.2 (2.5 26.2)*	Correlation between hsCRP, DBP, and bilirubin in mild preeclampsia and correlation between hsCRP, intrauterine fetal demise, fetal birth weight, labor intervention, uterine artery doppler changes, SBP, DBP, serum ALT, AST, creatinine, bilirubin, urea, hemoglobin and platelet levels in severe preeclampsia. No correlation between age, mode of delivery, and gestational age.
Chen et al., 2018 [17]	2.1±1.2	3.3±0.7	5.4±1.6	Correlation between hsCRP and severity of preeclampsia, no correlation with age, parity, and gestational age. No correlation between age of patient, parity, and gestational age.
Bansal et al., 2018 [18]	4.50 ± 1.09	9.06 ± 1.20	12.22 ± 1.93	Correlation between hsCRP and mean arterial pressure.
Jannesari and Kazemi, 2017 [1]	5.44 ± 3.94	6.70 ± 5.06	8.99 ± 7.27	Correlation between hsCRP, SBP, DBP, fetal birth weight, serum ALT, AST, LDH, hemoglobin, and creatinine. No correlation between age and gestational age of patient and blood platelet count.
Farzadnia et al., 2013 [19]	6.7±2.0	9.2±7.1	12.8±7.3	Correlation between hsCRP, fetal birth weight, blood pressure, serum ALT, and AST. No correlation between age of patient, serum bilirubin, creatinine, hemoglobin, and platelets.
Kashanian et al., 2013 [20]	3.6 ± 2.3	7.06 ± 2.6		Correlation between hsCRP, age of patients, SBP, DBP, fetal birth weight, and serum platelets. No correlation between gestational age and hemoglobin of patients.
Gandevani et al., 2012 [21]	2.5 ± 2.72	7.2 ± 2.2	9.4 ± 3.95	Correlation between serum hsCRP levels of patients. No correlation between age and parity of patients in study groups.
Bargale et al. 2011 [22]	1.216 ± 0.552	2.941 ± 0.390	4.769 ± 0.807	Correlation between hsCRP, SBP, and DBP of patients.
Ertas et al., 2010 [23]	5.8 ± 4.2	9.6 ± 7.1	23.4 ± 16.5	Correlation between hsCRP, SBP, DBP, fetal birth weight, fetal growth restriction, and adverse maternal outcomes. No correlation between age, gestational age, and parity of patients.
Tavana et al., 2010 [24]	2.64 ± 1.78	3.12 ± 1.31	3.43 ± 0.97	No correlation between serum hsCRP levels of patients.
Kumru et al., 2006 [25]	3.9 ± 2.5	9.5 ± 0.8		Correlation between hsCRP levels, SBP, DBP, fetal birth weight, serum creatinine, ALT, AST, LDH, and hemoglobin.
hsCRP levels in study (mean±SD)	Normal hsCRP	Raised hsCRP		Statistical inference
Present study	2.31 ± 0.45	5.85 ± 1.82		Positive correlation between hsCRP, SBP, DBP, serum bilirubin, ALT, AST, LDH, PT, INR, fetal ultrasound Doppler studies, fetal growth restriction, HDP, mode of delivery, and fetomaternal complications. Negative correlation between age, parity, serum hemoglobin, platelet count, and liquor index. No correlation between serum total proteins, creatinine, and urea.

## Conclusions

There is a statistically significant correlation between maternal serum hsCRP levels and the development of obstetric complications, leading to adverse fetomaternal outcomes. There is a positive correlation between maternal serum hsCRP levels and the severity of HDP. An increase in serum hsCRP levels also leads to increased incidence of labor interventions in patients with HDP. Thus, the present study suggests that patients having raised hsCRP levels have a poorer fetomaternal outcome and thus, must be used as a prognostic biomarker to identify high-risk cases before the onset of severe disease or adverse complications. It can also aid in prompt management and vigilance in patient care.
